# Protective effects of cell-free supernatants from a kefir-derived microbial consortium against multidrug-resistant *Candida glabrata* associated with vulvovaginal candidiasis

**DOI:** 10.1093/mmy/myag073

**Published:** 2026-07-10

**Authors:** Angela Maione, Marianna Imparato, Lea Di Massa, Annalisa Buonanno, Luigi Cirillo, Marco Guida, Elisabetta de Alteriis, Emilia Galdiero

**Affiliations:** Department of Biology, University of Naples Federico II, 80126 Naples, Italy; Department of Biology, University of Naples Federico II, 80126 Naples, Italy; Department of Biology, University of Naples Federico II, 80126 Naples, Italy; Department of Biology, University of Naples Federico II, 80126 Naples, Italy; Division of Urology, Asl Napoli3 Sud, 'San Leonardo' Hospital, 80053 Castellammare di Stabia, Italy; Department of Biology, University of Naples Federico II, 80126 Naples, Italy; Department of Biology, University of Naples Federico II, 80126 Naples, Italy; Department of Biology, University of Naples Federico II, 80126 Naples, Italy

**Keywords:** *Candida glabrata*, biofilm, postbiotics, wound healing, vulvovaginal candidiasis

## Abstract

Vulvovaginal candidiasis (VVC) caused by non-*albicans Candida* species, particularly *Candida glabrata*, is increasingly associated with treatment failure due to intrinsic or acquired resistance to antifungal drugs. In this study, we explored the anticandidal activity of a postbiotic cell-free supernatant (CFS) obtained from a microbial consortium isolated from homemade kefir (*Lactococcus lactis* subsp. *hordniae, Lactococcus lactis* subsp. *lactis*, and *Pichia kudriavzevii*) against one reference strain and two multidrug-resistant clinical isolates of *C. glabrata*. Antifungal susceptibility assays showed a fungistatic effect of CFS (Minimum Inhibitory Concentration [MIC] ≥ 500 µg/ml). Pre-treatment with sub-MIC concentrations (100 µg/ml) of CFS significantly reduced *C. glabrata* planktonic growth and cell surface hydrophobicity, as well as A-431 vaginal epithelial cell damage. Although total biofilm biomass of *C. glabrata* was not markedly affected by pre-treatment, metabolic activity was significantly decreased, together with downregulation of key virulence genes (*ALS3, ERG11*, and *FKS1*). Subsequently, we explored the effect of the CFS on the inhibition and eradication of biofilms derived from pretreated cells, showing a more marked effect compared to biofilms from untreated cells. Furthermore, in pre-treated fungal cells, the CFS almost completely abolished invasive capacity, reducing invasion in epithelial cell model and restored wound healing capacity following infection. Additionally, CFS modulated the host inflammatory response by reducing the expression of pro-inflammatory cytokines in A-431 cells. Overall, the kefir-derived postbiotic attenuated major virulence traits of *C. glabrata* and promoted epithelial protection, providing preliminary evidence supporting its potential as a complementary approach for VVC prevention and management.

## Introduction

Vulvovaginal candidiasis (VVC) is a medical condition that affects up to 75% of women of reproductive age at least once during their lives. Additionally, 5%–8% of these women go on to develop recurrent vulvovaginal candidiasis, characterized by three or more symptomatic episodes annually.

While *Candida albicans* has long been recognized as the primary cause of VVC, in recent decades, infections caused by non-*albicans Candida* (NAC) species have been increasingly reported. Among these, *Candida glabrata* (also classified as *Nakaseomyces glabrata*)^1^ is now one of the most frequently isolated species in NAC-VVC. VVC caused by *C. glabrata* is particularly difficult to treat due to its intrinsic or acquired reduced susceptibility to commonly used antifungal agents, increasing the risk of chronic or recurrent infections.^2,3^

Unlike *C. albicans*, which has a broad array of virulence factors, *C. glabrata* relies on a smaller but highly effective set of pathogenic traits. Adhesion to epithelial surfaces and biofilm formation are key factors contributing to persistence, antifungal tolerance, and immune evasion.^4,5^

Growing evidence suggests that the vaginal microbiota plays an important role in modulating susceptibility to VVC. *Lactobacillus*-dominated communities are generally associated with vaginal health, whereas dysbiosis may favor fungal overgrowth. Probiotic lactobacilli exert antimicrobial activity through multiple mechanisms, including competition for epithelial adhesion sites, co-aggregation, secretion of antimicrobial compounds, immunomodulation, and maintenance of a low vaginal pH via lactic acid production.^6–8^

Recently, yeasts have also been proposed as potential probiotic or biotherapeutic agents.^9^ Regarding VVC, at least one probiotic strain, *Saccharomyces cerevisiae* CNCM I-3856, is already commercially available in oral form and has demonstrated the ability to migrate from the gut to the vaginal environment.^10^ Several experimental studies have demonstrated that yeast-derived probiotics can interfere with *Candida* virulence by reducing adhesion, biofilm formation, epithelial damage, and by modulating the expression of pathogenicity-related genes.^11–15^

On the other hand, comparatively little attention has been devoted to postbiotics, defined as preparations of non-viable microorganisms and/or their metabolic products that confer health benefits to the host. Unlike probiotics, postbiotics eliminate the risks associated with the administration of live microorganisms while retaining biological activity through complex mixtures of microbial metabolites. Despite their growing interest, the effects of postbiotics in fungal infections, and particularly in NAC-associated VVC, remain poorly characterized.^16,17^

Recent studies have reported beneficial effects of *Lactobacillus*-derived postbiotics for vaginal health, mainly in the context of bacterial vaginosis.^18–20^

However, most available data focus on postbiotics derived from single microbial strains, and information on postbiotics produced by complex microbial consortia is still lacking. Therefore, the present work should be considered a preliminary, exploratory investigation aimed at providing initial insights into the biological effects of a multi-species–derived postbiotic.

Our previous work demonstrated that the cell-free supernatant (CFS), obtained from a defined kefir-derived microbial consortium of *Lactococcus lactis* subsp. *hordniae, Lactococcus lactis* subsp. *lactis*, and the yeast *Pichia kudriavzevii*, exhibited antimicrobial, antibiofilm, and antioxidant activities *in vitro*, as well as safety *in vivo* using the *Galleria mellonella* model.^21,22^ Furthermore, the same CFS was used as postbiotic supplements in the diets of mussels exposed to heavy metals in aquaculture environments.^23^

This study provides preliminary *in vitro* evidence on the effects of our CFS on *C. glabrata*–host cell interactions relevant to VVC, using an epithelial cell line. The first step involved the assessment of possible changes in specific virulence factors of *C. glabrata* by pre-incubating the yeast cells with CFS at sub-MIC (Minimum Inhibitory Concentration) concentration. Then, the pretreated cells were evaluated for the potential reduction in fungal proliferation, ability to form biofilm *in vitro*, the expression levels some biofilm associated genes, as well as enhancement of epithelial cell resistance to infection using the A-431 vaginal epithelial cell model. Then, to determine whether these CFS-induced changes on *Candida* cells persisted over time, the effect of a further exposure to the CFS was evaluated on the capacity to form biofilms and adhere/invade A-431 epithelial cells. Finally, the levels of some inflammatory cytokines in cells exposed to *C. glabrata* cells pre-treated or not were evaluated.

## Materials and methods

### Microbial strains and growth conditions

The reference strain *Candida glabrata DSM* 11226, obtained from the German Collection of Microorganisms and Cell Cultures (DSMZ), and two *C. glabrata* AO34, *C. glabrata* RCPF 1398 (C4, C15) isolated from VVC patients, were employed.^21,24^ Strains had been stored in frozen stocks at −80°C in Sabouraud Dextrose Broth (Thermo Fisher Scientific Inc., Waltham, MA, USA) supplemented with 15% glycerol. After thawing, the fungi were grown in Yeast Extract–Peptone–Dextrose broth (YPD) (Thermo Fisher Scientific Inc., Waltham, MA, USA) and incubated at 37°C under aerobic conditions for 24 h. Fungi in the exponential growth phase were used in the experiments.

### Preparation of Postbiotics CFS

The preparation of the postbiotic CFS was performed as previous described.^21^ Briefly, overnight cultures of *Lactococcus lactis* subsp. *hordniae, L. lactis* subsp. *lactis*, and *Pichia kudriavzevii* were co-inoculated in fresh De Man, Rogosa, and Sharpe (MRS, Oxoid Ltd., Basingstoke, UK) broth (1 × 10^3^ cells/ ml ratio 1:1:1), and incubated anaerobically at 37°C for 48 h. Supernatant was collected by centrifugation, filtered through 0.22 µm membranes. Sterility of the filtered CFS was assessed by plating 100 μl aliquots onto MRS and Sabouraud Dextrose Agar supplemented with chloramphenicol (Sigma-Aldrich, St. Louis, MO, USA). Plates were incubated at 37°C for 24–48 h to confirm the absence of viable microorganisms. CFS was lyophilized, resuspended in 1 phosphate-buffered saline (PBS), and pH-adjusted to 7.0 before use.

### Antifungal susceptibility testing

Antifungal susceptibility to CFS and azoles (itraconazole, ketoconazole, and fluconazole) was assessed according to CLSI M27-A3 guidelines.^25,26^ Fungal suspensions (1 × 10^3^ CFU/ml) were exposed to serial dilutions of CFS (5–500 μg/ml) or drug (0.125–64 μg/ml) in 96-well plates and incubated at 37°C for 24 h. MIC was determined spectrophotometrically (OD₅₉₀) as ≥ 80% growth inhibition, using a Varioskan LUX multimode microplate reader. For CFS, the minimum fungicidal concentration (MFC) was evaluated by plating aliquots from wells showing no visible growth onto Sabouraud dextrose agar (SAB, Thermo Fisher Scientific Inc., Waltham, MA, USA) and defined as ≥ 99.9% reduction in viability.

### A-431 epithelial cells

The human A-431 epithelial cell line was used as a preliminary *in vitro* epithelial model to investigate fungal–host interactions relevant to VVC. A-431 cells lobtained from the Leibniz Institute DSMZ–German Collection of Microorganisms and Cell Cultures (Braunschweig, Germany), were cultured in Dulbecco’s Modified Eagle’s Medium supplemented with 2 mM L-glutamine, 1% penicillin–streptomycin, and 10% heat-inactivated fetal bovine serum (Sigma Aldrich Co., St. Louis, MO, USA), and incubated at 37°C in a 5% CO_2_. Cells were subcultured at confluence of about 70%–80% using 0.25% trypsin–EDTA (ethylenediaminetetraacetic acid, Sigma Aldrich Co., St. Louis, MO, USA) and seeded at 1 × 10³ cells/well for experiments.

### Pre-treatment assay and growth kinetics

*<?tight ?>Candida glabrata* cells (DSM, C4, and C15) at concentration of 1 × 10⁶ cells/ml were incubated in YPD medium supplemented with CFS (100 µg/ml) for 24 h at 37°C under shaking conditions. Control cells received an equivalent volume of PBS. After incubation, cells were collected by centrifugation and washed twice with PBS, and resuspended in fresh YPD medium and used for subsequent experiments.

To determine whether CFS pre-treatment influenced subsequent planktonic growth, growth kinetics were evaluated for both untreated (CG-NT) and CFS pre-treated (CG-PT) cells. Planktonic growth was monitored for 24 h (OD₅₉₀) starting from initial OD of 0.1.

### Cell surface hydrophobicity assay

Cell surface hydrophobicity (CSH) of *C. glabrata* cells, pre-treated and untreated, was measured in a water-hydrocarbon two-phase assay.^27^*Candida glabrata* suspensions were adjusted to OD₆₀₀ of 0.40–0.50 (A₀) and mixed with n-hexadecane, vortexed vigorously for 60 s, and allowed to separate for 30 min at 30°C (A_1_). Hydrophobicity was calculated as:


\begin{eqnarray*}
\textit{Hydrophobicity}\,\left( \% \right) = \left[ {1 - \left( {{{A}_1}/{{A}_0}} \right)} \right] \times 100
\end{eqnarray*}

and categorized as low (0%–35%), moderate (36%–70%), or high (71%–100%).

### Lactate dehydrogenase release assay

Epithelial cell damage was assessed by measuring Lactate Dehydrogenase (LDH) release from A-431 cells infected with CG-NT or CG-PT (multiplicity of infection (MOI) of 5:1 for 24 h using a commercial LDH kit (Sigma-Aldrich), according to the manufacturer’s instructions.

### Biofilm formation and qualification

Biofilm formation was evaluated after 4, 24, and 48 h in 96-well plates using CG-NT and CG-PT (1 × 10⁶ cells/ml). Biofilm formation was evaluated by inoculating 200 µl of 1 × 10^6^ cells/ml suspension into 96-well flat-bottom polystyrene microtiter plates. The plates were incubated at 37°C for the selected experimental times, without agitation. After, non-adherent cells were removed by washing the wells twice and biofilm formation ability was evaluated in terms of total biomass and metabolic activity. Total biofilm biomass was quantified using crystal violet staining.^28^ Biofilm metabolic activity was assessed by means of the XTT [2,3-bis(2-methoxy-4-nitro-5-sulphophenyl)-2H-tetrazolium-5-carboxanilide] reduction assay.^29^

### qRT-PCR analysis

RNA from 24 h biofilms was extracted using Direct-zol™ RNA Miniprep Plus Kit. cDNA was synthesized using a Bio-Rad reverse transcription system. qRT-PCR was performed using SensiFAST™ SYBR Green Master Mix in a final reaction volume of 10 µl on an AriaMx Real-Time PCR System. Primer sequences are provided in Supplementary Table 1. Reactions were carried out with an initial polymerase activation step at 95°C for 2 min, followed by 40 amplification cycles including denaturation at 95°C for 5 s, annealing at 60°C for 10 s, and extension at 72°C for 5 s. A melting curve analysis was performed at the end of each qRT-PCR run to verify amplicon specificity. Amplification efficiencies for fungal primer pairs were determined from standard curves generated using serial cDNA dilutions, and the corresponding representative slope, linearity (*R*²), and efficiency values are reported in Supplementary Table 2. The individual melting curves are provided in Supplementary Figure 1. Data were acquired using Agilent Aria software (v1.7). Relative gene expression was calculated using the Pfaffl method and normalized to the *ACT1* reference gene using Relative Expression Software Tool (REST, v1.9.12).^30,31^

### Biofilm inhibition and eradication

For inhibition assays, CG-NT and CG-PT were incubated with CFS (75–250 µg/ml) during biofilm development in Roswell Park Memorial Institute (RPMI 1640) for 24 h at 37°C. For eradication assays, 24 h pre-formed biofilms were treated with CFS (75–250 µg/ml) for an additional 24 h at same temperature. Residual biomass was quantified by crystal violet staining.

### Adhesion and invasion assays on A-431 vaginal epithelial cells

Adhesion and invasion assays were performed on A-431 monolayers as previously described^32^ using CG-NT and CG-PT (MOI 5:1) in the presence of CFS (250 µg/ml). For the adhesion assay, infected monolayers were incubated for 2 h and then carefully washed to remove non-adherent fungal cells. After lysis of epithelial cells, adherent yeasts were quantified by CFU enumeration. For the invasion assay, infected monolayers were incubated for 6 h to allow fungal internalization. Thereafter, extracellular non-internalized yeasts were eliminated by treatment with amphotericin B (1 µg/ml), and epithelial cells were lysed to recover and quantify internalized fungal cells by CFU enumeration.

### Wound healing assay

The wound healing assay was performed using a scratch model to evaluate epithelial cell migration following *C. glabrata* infection. Confluent A-431 monolayers were mechanically scratched and subsequently infected with CG-NT or CG-PT cells (MOI 5:1). After removal of non-adherent fungi, cells were treated with CFS (250 µg/ml) and incubated for 24 h. Wound closure was monitored by image acquisition and quantified as the percentage of area reduction compared to the initial wound size.

### A431 cell treatments and inflammatory gene expression analysis (qPCR)

The *Candida glabrata* clinical isolate C15 (CG-NT and CG-PT) was used to infect A431 epithelial cells to evaluate the expression of inflammatory cytokines (IL-6, IL-8, and TGF-β). Cells were infected (MOI 5:1) for 2 h and subsequently treated with CFS (250 µg/ml) for an additional 2 h. Primer sequences are reported in Supplementary Table 3. Expression of *IL-6, IL-8*, and *TGF-β* was analyzed by qPCR and normalized to *β-actin* using the 2^−ΔΔCt^ method.^33^ qPCR reactions were performed with an initial polymerase activation step at 95°C for 2 min, followed by 40 amplification cycles consisting of denaturation at 95°C for 5 s, annealing at 65°C for 10 s, and extension at 72°C for 20 s. A melting curve analysis was performed at the end of each qRT-PCR run to verify amplicon specificity. Amplification efficiencies for the human primer pairs were determined from standard curves generated using serial cDNA dilutions, and the corresponding representative slope, linearity (*R*²), and efficiency values are reported in Supplementary Table 4. The individual melting curves are provided in Supplementary Figure 2.

### Statistical analysis

All experiments were performed in triplicate and repeated independently at least three times. Technical triplicates were averaged and not treated as independent replicates. Data are presented as mean ± standard deviation (SD). Statistical analyses were carried out using GraphPad Prism, version 8 (GraphPad Software, La Jolla, CA, USA). Comparisons between two groups were performed using Student’s *t*-test, while multiple comparisons were conducted by one-way or two-way analysis of variance followed by Tukey’s post hoc test. Given the limited number of independent biological replicates, formal normality testing was not performed. Differences were considered statistically significant at *P* < .05.

## Results

### Antifungal susceptibility

The antifungal susceptibility profiles of the *Candida glabrata* DSM reference strain and the two clinical isolates (C4 and C15) are summarized in Table 1. The DSM strain displayed susceptibility to all tested azoles, with MIC values of 1 μg/ml for Itraconazole (ITZ), 0.5 μg/ml for Ketoconazole (KET), and 2 μg/ml for Fluconazole (FLC). In contrast, both clinical isolates exhibited reduced susceptibility and clear resistance patterns. *Candida glabrata* C4 and C15 were resistant to itraconazole (MIC > 64 μg/ml) and fluconazole (MIC > 64 μg/ml) and showed intermediate susceptibility to ketoconazole (MIC 8–16 μg/ml).

**Table 1.** Minimum inhibitory concentration (MIC) of azoles against *Candida glabrata* strains determined using the Clinical and Laboratory Standards Institute (CLSI) broth microdilution method.

Strain	Itraconazole (ITZ)	Ketoconazole (KET)	Fluconazole (FLC)	Phenotypic profile
*Candida glabrata* DSM	1 µg/ml	0.5 µg/ml	2 µg/ml	Susceptible
*Candida glabrata* C4	> 64 µg/ml	8 µg/ml	> 64 µg/ml	Resistant to ITZ and FLC; intermediate to KET
*Candida glabrata* C15	> 64 µg/ml	16 µg/ml	> 64 µg/ml	Resistant to ITZ and FLC; intermediate to KET

The inhibitory effect of CFS on *C. glabrata* strains was evaluated by determining MIC values.

The results showed that the MIC values of all three strains of *C. glabrata* was 500 μg/ml for collection strain and above 500 μg/ml for the two clinical isolates. Results suggested that CFS can moderately inhibit the growth of all tested *C. glabrata* strains. In addition, MFC values were determined for all three microorganisms, and in all cases the MFC was greater than 500 μg/ml, indicating that the CFS exhibited a fungistatic rather than fungicidal effect.

A sub-MIC concentration of CFS 100 μg/ml was selected to carry out across the pre-treatment subsequent virulence experiments.

### Planktonic curve growth of *C. glabrata* cells pre-treated with CFS

**Figure 1.**
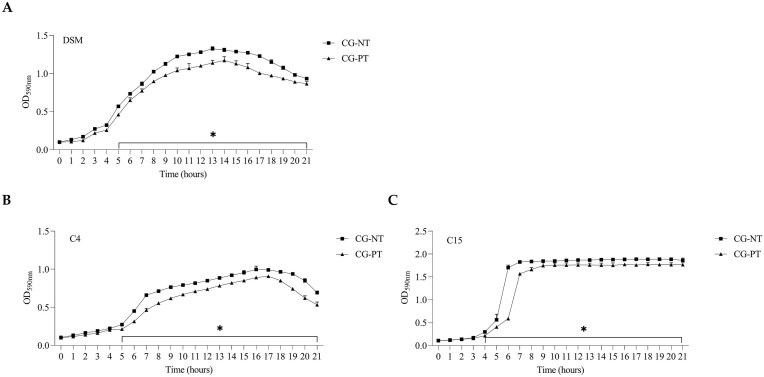
Growth curves of *Candida glabrata* strains not treated (CG-NT) and pre-treated with CFS 100 µg/ml (CG-PT). (A) *Candida glabrata* DSM reference strain, (B) clinical isolate *C. glabrata* C4, and (C) clinical isolate *C. glabrata* C15. Growth kinetics were monitored over 24 h by measuring optical density at 590 nm (OD_590_) at regular intervals. * *P* < .05 (Tukey’s test).

The effect of pre-treatment with CFS derived from the multispecies consortium on the planktonic growth *C. glabrata* cells of the three strains (DSM, C4, and C15) was evaluated. Pretreated *Candida* cells (CG-PT) were suspended and cultured in YPD medium, in parallel with the non-treated counterpart (CG-NT). As shown in Figure 1 (panels A–C) the growth kinetics of control (non-treated) *C. glabrata* cells (CG-NT) and pre-treated cells (CG-PT) were monitored over approximately 20 h by measuring optical density (OD_590_). All strains exhibited a typical growth profile, characterized by an initial lag phase followed by an exponential increase in cell density up to a stationary phase, with the clinical isolate C15 showing the highest growth rate and cell density. However, for all three strains tested, CG-PT displayed a reduced growth rate compared with CG-NT and reached a lower stationary-phase plateau than the corresponding CG-NT, suggesting that the 24-h pretreatment with the CFS negatively affected the proliferative capacity of *C. glabrata*.

### CFS pre-treatment decreases cell surface hydrophobicity of *C. glabrata*

**Figure 2.**
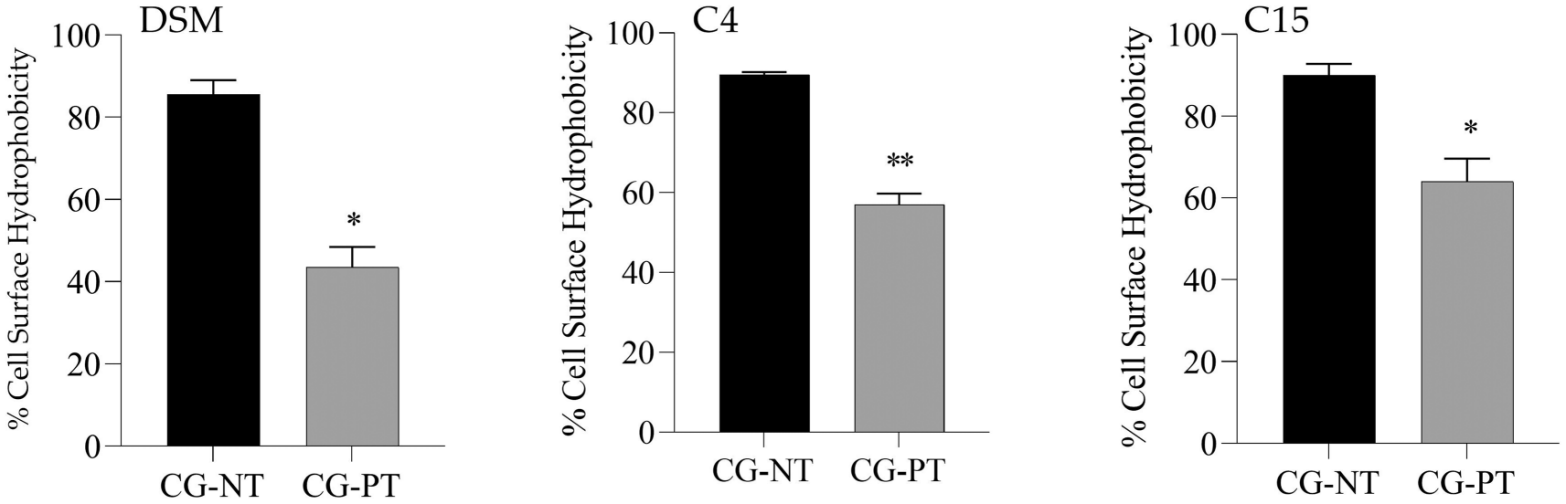
Effect of CFS pre-treatment on the cell surface hydrophobicity of *Candida glabrata* DSM, C4, and C15. The cell surface hydrophobicity of *C. glabrata*, as measured in a water-hydrocarbon two-phase assay in test tubes at OD value of 600 nm. Data are the mean ± SD (*n* = 3).* *P* < .05, ** *P* < .01, versus the control group (*t*-test).

Among virulence factors, the assessment of CSH is particularly important, as a positive correlation exists between CSH and adhesion. In this study, we evaluated whether pre-treatment of *C. glabrata* cells with CFS could influence CSH compared with untreated cells. Following CFS exposure, a marked decrease in the upper organic phase was detected, reflecting a reduction in CSH (Fig. 2). These results indicate that CFS treatment moderately modifies the surface properties of *C. glabrata* cells compared with untreated controls.

### CFS pretreatment reduces cytotoxicity (LDH release)

**Figure 3.**
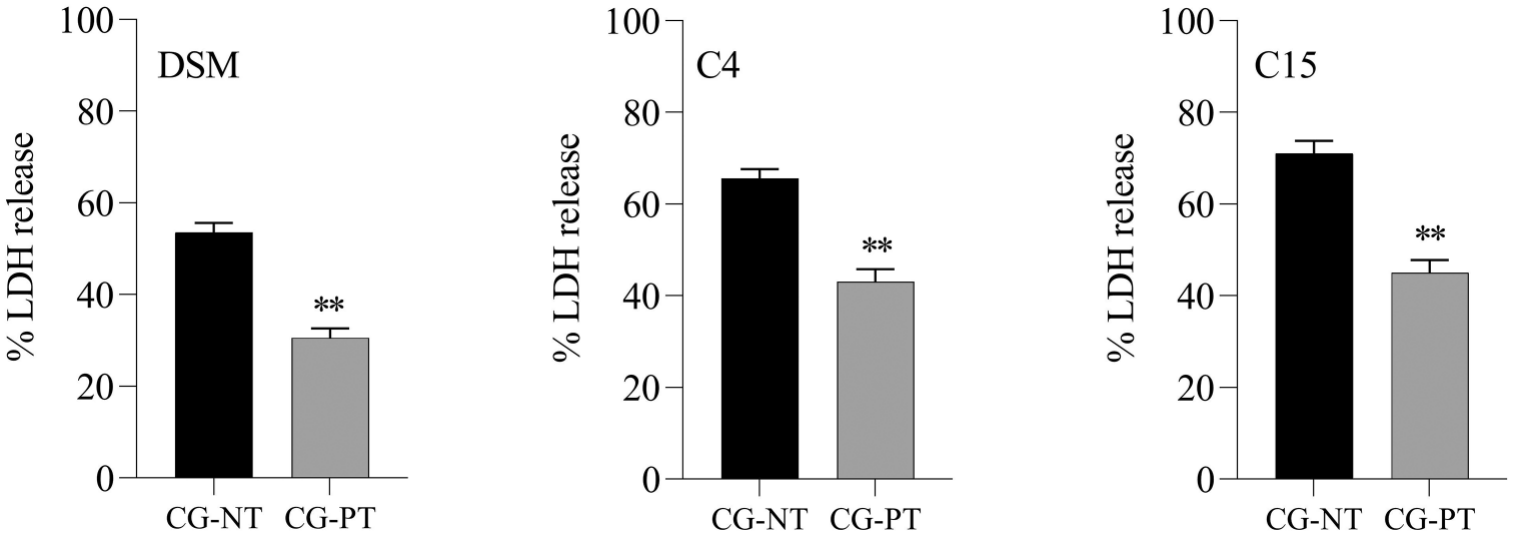
Percentage of LDH release of A-431 cells infected with *Candida glabrata* CG-NT or CG-PT (MOI 5:1); (A) DSM, (B) C4; and (C) C15. ** *P* < .01 (*t*-test).

Since CFS pre-treatment of the three strains was designed to reduce their virulence, we performed an LDH assay to evaluate its protective properties. A431 cells were infected with CG-NT and CG-PT. The results of these experiments indicated that infection with CG-NT induced cytotoxicity of about 50% for DSM and > 60% for C4 and C15. In contrast, pre-exposure significantly reduced (*P* < .01) *C. glabrata*-induced cytotoxicity, with a reduction of about 25% for all three strains (Fig. 3). Thus, the pre-treatment clearly contributed to reducing cytotoxicity of *C. glabrata* in vaginal epithelial cells.

### Effect of CFS-pretreatment on biofilm formation

**Figure 4.**
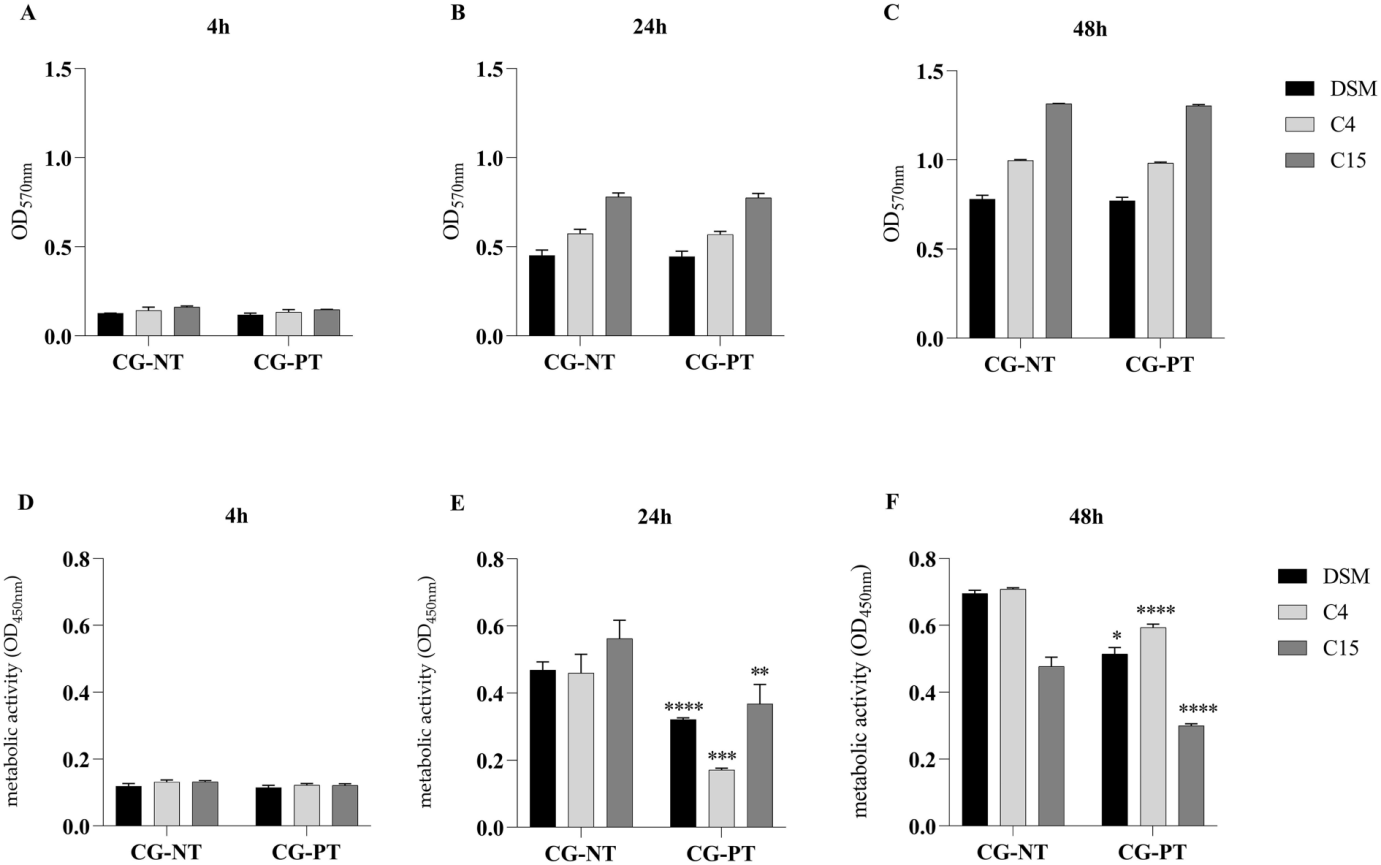
Quantification of biofilm biomass after 4 h (A), 24 h (B), and 48 h (C) of incubation, measured at OD₅₉₀ nm using crystal violet staining. Biofilm metabolic activity measured at OD₄_5_₀ nm by XTT reduction assay at 4 h (D), 24 h (E) and 48 h (F). Data are expressed as mean ± SD from three independent experiments performed in triplicate. Asterisks indicate statistically significant differences compared to CG-NT (*P < .05, **P < .01, ***P < .001, ****P < .0001, Tukey’s test).

In the present study, we analysed the biofilm-forming ability of *C. glabrata* CG-PT strains in comparison with their respective CG-NT controls over a 48 h period (Fig. 4). The results showed that during the initial adhesion phase (0–4 h) (Fig. 4, panel A), no statistically significant differences were observed between NT and PT biofilms for any of the three strains, all of which exhibited a weak ability to form biofilms. Likewise, in the later stages of biofilm development (24–48 h) (Fig. 4, panels B and C), no significant differences were detected among the strains, which all displayed a strong capacity to form mature biofilms, as indicated by crystal violet quantification. Interestingly, the clinical isolate C15, either pretreated or not, consistently showed the highest biofilm-forming ability at all time points compared to the other strains. Although all three strains demonstrated a robust ability to form biofilms under both conditions, cell viability within the total biomass, assessed by XTT, was markedly reduced in all strains in the PT condition at both 24 and 48 h, as shown in Fig. 4 (panels C and D). A significant decrease in metabolic activity was detected in CG-PT samples compared with their NT counterparts. In particular, biofilms in the CG-PT condition exhibited lower viable biomass than those in the CG-NT condition for all strains, with an inhibition of metabolic activities of approximately 30% in *C. glabrata* DSM and greater than 50% in C4 and C15. A similar trend was observed at 48 h., with CG-PT biofilms from all three strains showing a 40% reduction in viable biomass compared to CG-NT.

**Figure 5.**
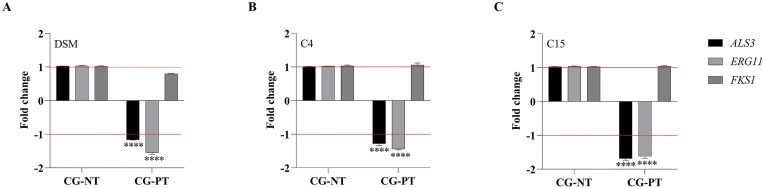
Expression analysis of selected genes (*ALS3, ERG11*, and *FKS1*) of *Candida glabrata* DSM 11226, C4, and C15 strains using real-time qPCR. Histograms represent the fold differences in the expression levels of the genes selected. Red lines show fold-change thresholds of -1 and + 1, respectively. * *P* < .05 indicates fold changes significantly different from untreated samples (*t*-test).

To assess whether CFS pre-treatment interferes with biofilm development through modulation of biofilm-associated gene expression, the transcriptional levels of genes involved in ergosterol biosynthesis (*ERG11*), β-1,3-glucan synthesis (*FKS1*), and adhesion (*ALS3*) were analyzed by qRT-PCR, as shown in Figure 5. In the reference strain (panel A), pre-treatment with CG-PT resulted in a marked downregulation of all three virulence genes, with the strongest effect observed for *ERG11* and *FKS1*, which exhibited a statistically significant decrease compared with untreated cells (*P* < .001). In contrast, CG-NT induced only minor and non-significant variations in gene expression. In clinical isolate C4 (panel B), a similar trend was observed. CG-PT significantly reduced the expression of *ALS3, ERG11*, and *FKS1*, whereas CG-NT caused only a slight, non-significant modulation of transcriptional levels. In clinical isolate C15 (panel C), pre-treatment with CG-PT again led to a significant downregulation of all three genes, with particularly strong suppression of *ALS3* and *ERG11*. CG-NT induced minimal changes also in this isolate.

Taken together, these findings support the hypothesis that CFS pretreatment can weaken key virulence attributes of *C. glabrata*, such as the biofilm formation capacity, highlighting the potential of CFS to counteract virulence traits associated with persistence and antifungal tolerance. This observation is consistent with the reduced hydrophobicity detected in pretreated cells.

### Effect of CFS on inhibition and eradication of biofilms formed by pre-treated *C. glabrata* cells

**Figure 6.**
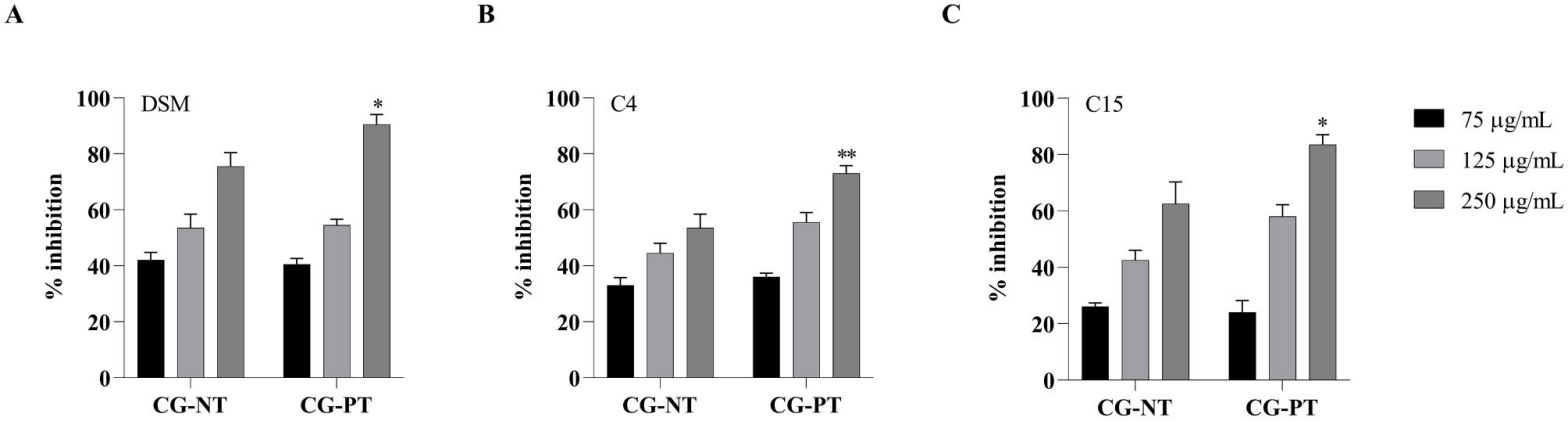
Percentage of biofilm inhibition of *Candida glabrata* non-treated (CG-NT) and pre-treated (CG-PT) after exposure to CFS (75, 125, and 250 µg/ml). (A) *Candida glabrata* DSM; (B) *C. glabrata* C4; and (C) *C. glabrata* C15. Bars represent mean ± standard deviation of three independent experiments expressed as percentage relative to untreated controls. Asterisks represent significant differences vs CG-NT (* *P* < .05, ** *P* < .01, Tukey’s test).

**Figure 7.**
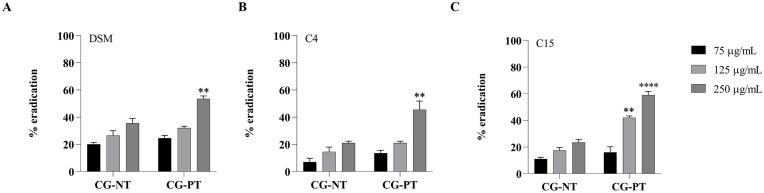
Percentage of biofilm eradication of *Candida glabrata* non-treated (CG-NT) and pre-treated (CG-PT) after exposure to CFS (75, 125, and 250 µg/ml). (A) *Candida glabrata* DSM; (B) *C. glabrata* C4; and (C) *C. glabrata* C15. Bars represent mean ± standard deviation of three independent experiments expressed as percentage relative to untreated controls. Asterisks represent significant differences vs CG-NT (* *P* < .01, **** *P* < .0001, Tukey’s test).

Once ascertained that the CFS pre-treatment affects some virulence traits of *C. glabrata*, we explored the effect of the CFS on the inhibition and eradication of biofilms derived from pretreated cells. So, the effect of CFS on the ability of untreated (CG-NT) and pre-treated (CG-PT) *C. glabrata* cells to form biofilms was evaluated through inhibition and eradication assays, using three different CFS concentrations (75, 125, and 250 µg/ml). As shown in Fig. 6 (A–C), CFS treatment resulted in a dose-dependent inhibition by CFS of biofilm formation in all tested isolates (*C. glabrata* DSM, C4, and C15). At the highest concentration, the inhibition rate reached approximately 90% for DSM-PT, 70% for C4-PT, and 85% for C15-PT compared to untreated controls. Significant differences were observed between CG-NT and CG-PT for all isolates, indicating that the pre-treatment affected the early stages of biofilm development. Conversely, in the biofilm eradication assay (Fig. 7, A–C), a different trend was observed. No significant differences in biofilm eradication were detected at the lower CFS concentrations. Pre-treated cells (CG-PT) exhibited a higher eradication rate than their non-treated counterparts, although this did not exceed 60% and was observed only at the highest concentration tested. For example, at 250 µg/ml, eradication reached approximately 50% for DSM, 40% for C4, and 60% for C15 in CG-PT samples, whereas CG-NT values remained lower (35%, 21%, and 25%, respectively). The difference was statistically significant (*P* < .05), particularly at 125 and 250 µg/ml. These findings suggest that pre-exposure to CFS may alter cell surface properties or biofilm architecture, increasing the susceptibility of *C. glabrata* biofilms to subsequent CFS treatment, and that developing biofilms are more sensitive than mature ones.

### Effect of CFS on adhesion and invasion of pre-treated *C. glabrata* in A-431 cells

**Figure 8.**
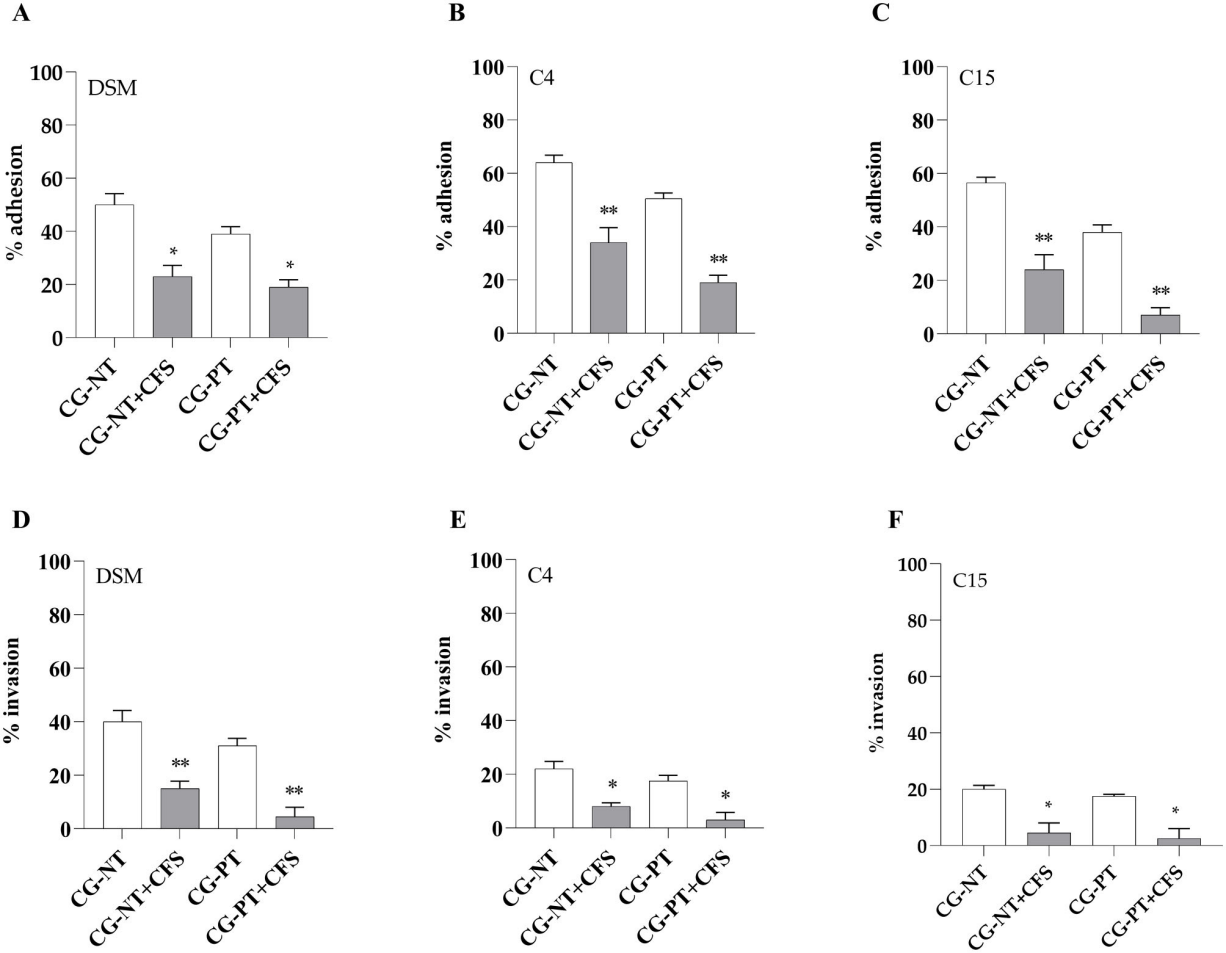
Effect of the CFS (250 µg/ml) on adhesion and invasion of *Candida glabrata* to A-431 epithelial cells. Panels A–C show the percentage of adhesion for *C. glabrata* DSM (A), C4 (B), and C15 (C) strains, while panels D–F show the corresponding invasion data for DSM (D), C4 (E), and C15 (F). Data are presented as mean ± SD of three independent experiments performed in triplicate. Asterisks represent significant differences vs untreated control (CG-NT or CG-PT) (*P < .05, **P < .01, ***P < .001, ****P < .0001, Tukey’s test).

Based on the obtained effects of CFS on biofilm formation, we further investigated its impact on *C. glabrata* (CG-NT and CG-PT)–vaginal epithelial cell interactions by assessing adhesion and invasion in A-431 cells.Only the concentration of 250 µg/ml was selected for performing the *in vitro* adhesion and invasion assays on A-431 vaginal epithelial cells. First of all, as reported in Figure 8, the three pre-treated strains were more impaired in their adhesive and invasive capacities, a result is in agreement to the overall impairment of the biofilm forming ability already shown. In the adhesion assay CFS reduced fungal attachment in CG-NT + CFS compared with untreated controls CG-NT from approximately 50% to 23%, for DSM, 64% to 34% and 56% to 24% for C4 and C15 respectively. A similar trend was observed CG-PT after CFS treatment (CG-PT + CFS) compared to their controls CG-PT, with adhesion decreasing from 38% to 18% for DSM, 50% to 19% for C4, and 38% to 7% for C15. (Fig. 8, panels A–C). Regarding the invasion assay, in the CG-NT + CFS condition, invasion was drastically reduced from 40% to 15% for DSM, from 22% to 8% for C4, and from 20% to 5% for C15. Remarkably, in pre-treated fungal cells CG-PT + CFS, CFS almost completely abolished invasive capacity, reducing invasion from 32% to 5% for DSM, from 17% to 3% for C4, and from 18% to as low as 2%–3% for C15 (Fig. 8, panel D–F). Taken together, these results demonstrated that although CFS exerted only moderate effects on *C. glabrata* growth, it significantly inhibited adhesion and invasion and protected A431 cells from damage caused by these yeasts in both pre-treated and non–pre-treated conditions.

### Effect of CFS on wound healing assay

**Figure 9.**
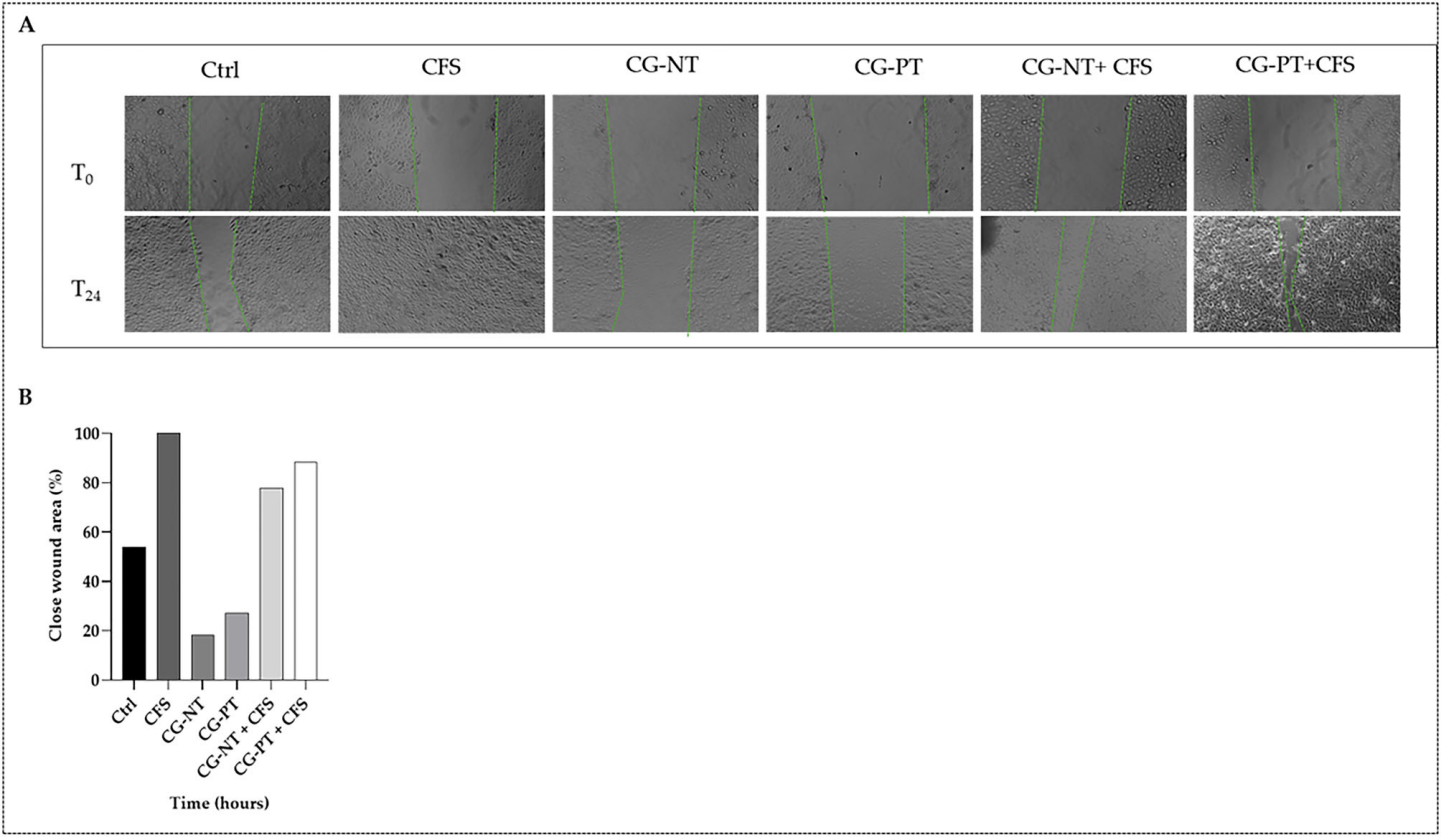
Wound healing assay of A-431 epithelial cells infected with *Candida glabrata* clinical isolate (C15) and treated with CFS (250 µg/ml).

The effect of *C. glabrata* infection, CFS pre-treatment and following CFS treatment on the migratory capacity of A-431 epithelial cells was evaluated using a wound healing assay (Fig. 9). After 2 h of infection with either non-treated (CG-NT) or pre-treated (CG-PT) *C. glabrata* cells, the monolayers were washed to remove non-adherent yeasts, and fresh medium containing CFS (250 µg/ml) was added for 24 h. At time 0, all groups showed a comparable wound area. After 24 h, control (uninfected) cells displayed near-complete wound closure, indicating normal migratory activity. In contrast, infection with *C. glabrata* significantly delayed wound closure in all strains tested, particularly in the CG-NT group, where a large open wound area persisted. Cells infected with *C. glabrata* CG-PT exhibited slightly improved recovery compared with CG-NT (Fig. 9A). Remarkably, the addition of CFS after infection restored the migratory capacity of A-431 cells, leading to a substantial reduction in the wound area compared with infected untreated samples. Quantitative analysis confirmed this observation, showing a wound closure rate of approximately 80% in the control group, 30%–40% in infected untreated cells, and up to 70% in infected cells treated with CFS (Fig. 9B).

### Effect of CFS on modulation of the expression level of proinflammatory and anti-inflammatory genes in *C. glabrata*-infected A-431 cells

**Figure 10.**
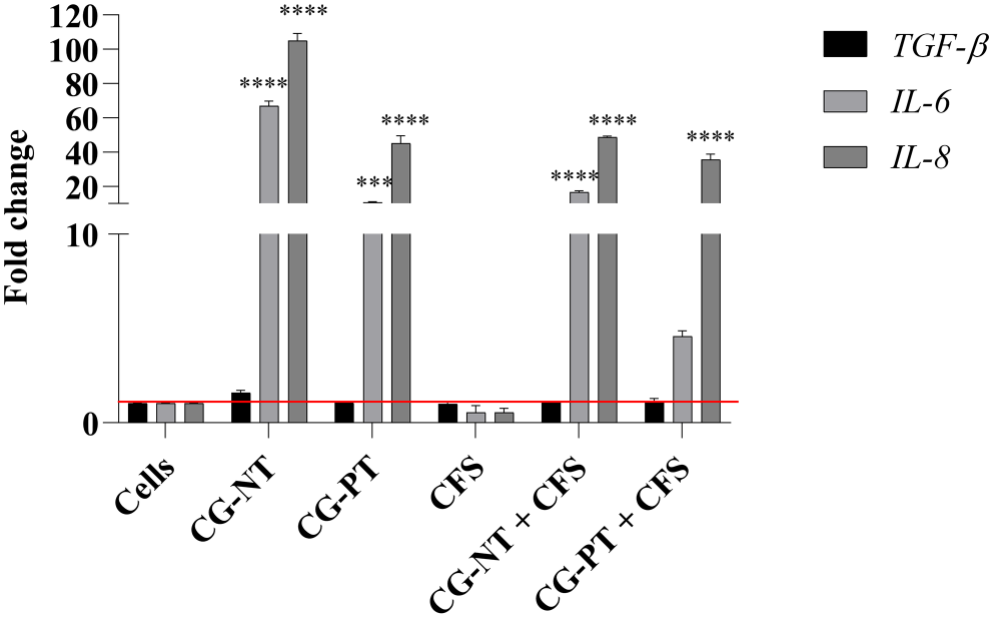
Relative gene expression levels of *TGF-β, IL-6*, and *IL-8* in cells exposed to CG-NT, CG-PT, CFS alone, and their combinations with CFS. Data are expressed as fold change relative to untreated control cells (Cells), normalized against the housekeeping gene. Data represent mean ± SD of three independent experiments. *** *P* < .001****, *P* < .0001.

To investigate the effects of CFS on the expression level of proinflammatory genes including *IL-6, IL-8*, and *TGF-β*, A-431 cells were treated with the clinical strains (C15) pre-treated or not. As shown in Figure 10, exposure to CG-NT resulted in a marked increase in the expression of both *IL-6* and *IL-8*, with levels significantly higher than those observed in control cells (*P* < .0001), indicating a strong pro-inflammatory response. A similar trend, although less pronounced, was observed in cells treated with CG-PT, which also showed a significant upregulation of both cytokines compared to the control condition. The addition of CFS markedly influenced the inflammatory profile. In particular, cells treated with CG-NT + CFS and CG-PT + CFS exhibited an overall reduction in *IL-6* and *IL-8* expression compared to the corresponding treatments without CFS, although values remained significantly higher than in control cells (*P* < .0001). These findings suggest a modulatory effect of CFS on the inflammatory response induced by CG treatments. In contrast, *TGF-β* expression remained consistently low across all experimental conditions, showing only minor variability and no substantial differences between treatments, indicating that CG treatments and CFS do not significantly affect the regulation of this anti-inflammatory cytokine in the tested cellular model.

## Discussion

The incidence of VVC caused by NAC species, particularly *Candida glabrata*, has constantly increased over recent decades and represents a growing clinical challenge.^34^ Despite its lack of hyphal morphogenesis, *C. glabrata* relies on adhesion, biofilm formation, and metabolic adaptation to establish persistent infections, highlighting the need for alternative therapeutic strategies aimed at attenuating virulence rather than exerting direct fungicidal pressure.^35^

It is well established that probiotics display pronounced strain-specific activities, and their biological effects may vary considerably even within the same species.^36^ Lactobacilli, dominant members of the healthy vaginal microbiota, contribute to vaginal homeostasis through competitive exclusion of pathogens, modulation of epithelial immune responses, and production of antimicrobial metabolites such as organic acids, hydrogen peroxide, and bacteriocins.^37^

In contrast, considerably less is known about the role of postbiotics, defined as non-viable microbial cells and/or their bioactive metabolites, in the prevention or treatment of VVC. Postbiotics offer several advantages over live probiotics, including improved stability, safety in immunocompromised individuals, and clearer standardization of bioactive components.^38^ Previous studies have demonstrated that postbiotic preparations can inhibit the growth and biofilm formation of *C. albicans*, although evidence regarding their effects on NAC species remains limited.^39–41^ Moreover, most available studies have focused on postbiotics derived from single microbial strains, whereas the biological activity of postbiotics produced by complex, multi-species consortia remains largely unexplored.

In the present study, we evaluated for the first time the antifungal and antivirulence potential of the CFS obtained from a kefir-derived microbial consortium against multidrug-resistant clinical isolates of *C. glabrata*. Our approach deliberately focused on sub-MIC concentrations of CFS to investigate whether postbiotic exposure could impair fungal virulence traits without exerting strong selective pressure on growth, in line with emerging antivirulence strategies. Importantly, the CFS was used as a whole postbiotic preparation, reflecting the combined activity of multiple microbial metabolites rather than the action of a single defined compound.

Our data demonstrate that CFS exerted a moderate fungistatic effect on *C. glabrata* planktonic growth, consistent with previous reports describing postbiotics as antivirulence rather than fungicidal agents.^42^ Notably, CFS pretreatment significantly reduced CSH, a key determinant of fungal adhesion to epithelial surfaces and abiotic substrates. This reduction likely represents an early mechanism through which CFS interferes with fungal pathogenicity.

Although CFS pretreatment did not significantly alter total biofilm biomass, it led to a marked reduction in biofilm metabolic activity, suggesting the development of structurally preserved but metabolically impaired biofilms. Given the role of metabolically active biofilms in persistence and antifungal tolerance, this functional impairment may be clinically relevant. Biofilm biomass and metabolic activity are known to reflect distinct biological features rather than overlapping outcomes.^43^ In line with these observations, transcriptional analysis revealed reduced expression of genes associated with adhesion (*ALS3*), cell wall integrity (*FKS1*), and ergosterol biosynthesis (*ERG11*), indicating that postbiotic exposure interferes with multiple pathways required for biofilm maturation and stability.

The antivirulence effects of CFS were further supported by host-pathogen interaction assays. Results were obtained using the A431 epithelial cell line .^44^ Both adhesion and invasion of *C. glabrata* were significantly reduced following CFS pretreatment and further exposure, with invasion being almost completely abolished, suggesting a cumulative weakening of fungal invasive capacity. Although A-431 cells represent a useful and reproducible preliminary epithelial model, they do not fully reproduce the vaginal mucosal environment, and further studies using more physiologically relevant models will be needed to confirm these findings.^45^ Our results are consistent with previous reports showing that probiotic-derived metabolites can interfere with *Candida*-host interactions by modulating fungal surface properties and epithelial recognition mechanisms.^46^

Epithelial protection was further supported by LDH release and wound healing experiments. CFS pretreatment significantly reduced epithelial cell damage induced by *C. glabrata*, while post-infection supplementation with CFS restored epithelial migratory capacity. Together, these data suggest a dual action of postbiotics, targeting both fungal virulence and epithelial resilience.

In VVC, proinflammatory cytokines are commonly produced in the vaginal tissue showing that inflammatory response appears to be important during their pathogenesis. The observed modulation of inflammatory gene expression, characterized primarily by reduced *IL-6* and *IL-8* levels in the presence of CFS, supports a potential immunomodulatory role of postbiotics.

Despite these promising findings, this study remains exploratory. Further validation using physiologically relevant vaginal epithelial models and *in vivo* systems will be required to confirm the translational relevance of these observations.

Collectively, our findings indicate that exposure of *C. glabrata* to probiotic-derived CFS results in a reduction of key virulence traits of key virulence traits, rendering yeast cells more susceptible to subsequent interventions. Rather than acting as a classical antifungal agent, the CFS appears to function as a virulence-weakening modulator, impairing adhesion, biofilm metabolic activity, epithelial invasion, and host cell damage. This strategy aligns with emerging concepts in antifungal therapy that emphasize disarming pathogens instead of eradicating them, thereby potentially reducing resistance development.^47^

Future studies should identify the bioactive components responsible for these effects and validate CFS efficacy and safety *in vivo* models of vaginal candidiasis. In particular, the impact of CFS on vaginal microbiota composition, mucosal immune responses, and its potential synergy with conventional antifungal agents warrants further investigation. From a translational perspective, standardized topical postbiotic formulations may represent a safe and innovative adjunctive strategy for the management of *C. glabrata*-associated VVC.

## Supplementary Material

myag073_Supplemental_File
