# Supplementary material for: Protective effects of cell-free supernatants from a kefir-derived microbial consortium against multidrug-resistant Candida glabrata associated with vulvovaginal candidiasis
Source: Med Mycol. 2026 Jul 10;64(7):myag073. doi: 10.1093/mmy/myag073 (PMC13411789; doi:10.1093/mmy/myag073)
Supplement: myag073_Supplemental_File [file myag073_supplemental_file.docx]

**Table S1.** Gene-specific primers used for real-time RT-PCR.

| Gene | Forward (5'→3') | Reverse (5'→3') |
| --- | --- | --- |
| *ACT1* | TTGCCACACGCTATTTTGAG | ACCATCTGGCAATTCGTAGG |
| *ERG11* | ATTGGTGTCTTGATGGGTGGTC | TCTTCTTGGACATCTGGTCTTTCA |
| *ALS3* | CTGGACCACCAGGAAACACT | GGTGGAGCGGTGACAGTAGT |
| *FKS1* | GTTGCAGTCGCTACATTGCTA | TAGCGTTCCAGACTTGGGAA |

**Table S2.** qRT-PCR amplification performance of the fungal primer pairs used in this study.

| Gene | Target | Slope | R² | Efficiency (%) |
| --- | --- | --- | --- | --- |
| ACT1 | *C. glabrata* | -3.38 | >0.990 | 98 |
| ERG11 | *C. glabrata* | -3.34 | >0.992 | 100 |
| ALS3 | *C. glabrata* | -3.44 | >0.988 | 96 |
| FKS1 | *C. glabrata* | -3.43 | >0.990 | 96 |


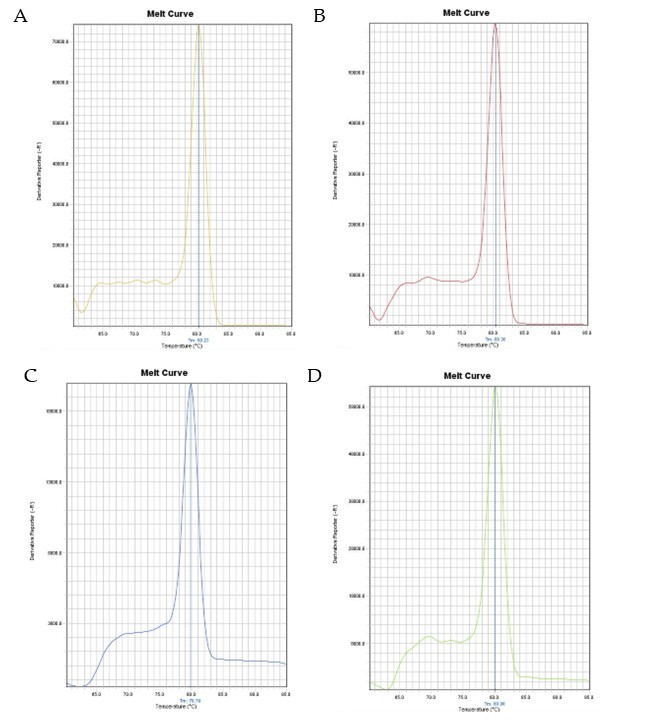


**Figure S1.** Melting curves for fungal targets; A) ACT1, B) ERG11, C) ALS3, D) FKS1.

**Table S3.** Primers used for qPCR in A431 cells

| Gene | Forward (5'→3') | Reverse (5'→3') |
| --- | --- | --- |
| *IL-6* | GTGATGCCCCAAGCTGAGA | CACGGCCTTGCTCTTGTTTT |
| *IL-8* | CTGGCCGTGGCTCTCTTG | GGGTGGAAAGGTTTGGAGTATG |
| *TGF-β* | CCCAGCATCTGCAAAGCTC | GTCAATGTACAGCTGCCGCA |
| *β-actin* | ATGTGGCCGAGGACTTTGATT | AGTGGGGTGGCTTTTAGGATG |

**Table S4.** qRT-PCR amplification performance of the human primer pairs used in this study.

| Gene | Target | Slope | R² | Efficiency (%) |
| --- | --- | --- | --- | --- |
| β-actin | Human | -3.34 | >0.995 | 100 |
| IL-6 | Human | -3.45 | >0.985 | 95 |
| IL-8 | Human | -3.49 | >0.985 | 94 |
| TGF-β | Human | -3.40 | >0.990 | 97 |


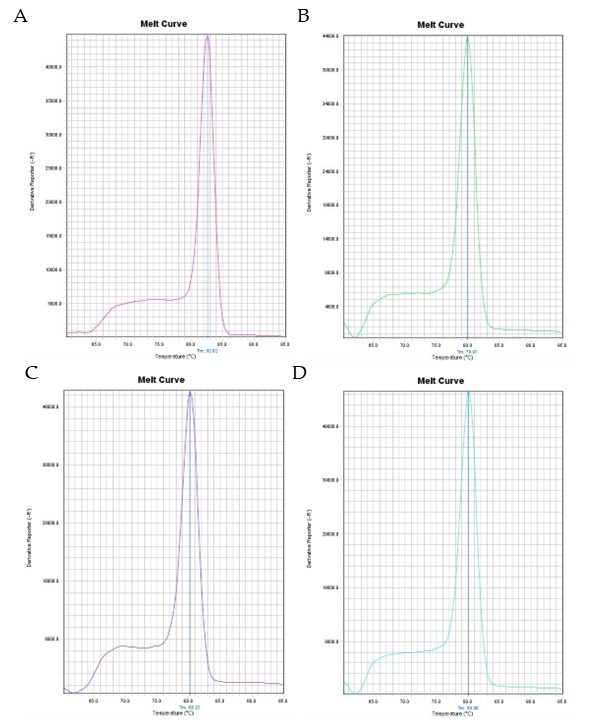


**Figure S2.** Melting curves for human targets; A) β-actin, B) IL-6, C) IL-8, D) TGF-β.
